# Regulation of carbamoylphosphate synthesis in *Escherichia coli*: an amazing metabolite at the crossroad of arginine and pyrimidine biosynthesis

**DOI:** 10.1007/s00726-018-2654-z

**Published:** 2018-09-20

**Authors:** Daniel Charlier, Phu Nguyen Le Minh, Martine Roovers

**Affiliations:** 1 Research Group of Microbiology, Department of Bio-engineering Sciences, Vrije Universiteit Brussel, Pleinlaan 2, 1050 Brussels, Belgium; 2 LABIRIS Institut de Recherches, Av. Emile Gryson 1, 1070 Brussels, Belgium

**Keywords:** Carbamoylphosphate synthase, Arginine biosynthesis, Tandem promoters, Transcription regulation, DNA remodeling, Allosteric control

## Abstract

In all organisms, carbamoylphosphate (CP) is a precursor common to the synthesis of arginine and pyrimidines. In *Escherichia coli* and most other Gram-negative bacteria, CP is produced by a single enzyme, carbamoylphosphate synthase (CPSase), encoded by the *carAB* operon. This particular situation poses a question of basic physiological interest: what are the metabolic controls coordinating the synthesis and distribution of this high-energy substance in view of the needs of both pathways? The study of the mechanisms has revealed unexpected moonlighting gene regulatory activities of enzymes and functional links between mechanisms as diverse as gene regulation and site-specific DNA recombination. At the level of enzyme production, various regulatory mechanisms were found to cooperate in a particularly intricate transcriptional control of a pair of tandem promoters. Transcription initiation is modulated by an interplay of several allosteric DNA-binding transcription factors using effector molecules from three different pathways (arginine, pyrimidines, purines), nucleoid-associated factors (NAPs), trigger enzymes (enzymes with a second unlinked gene regulatory function), DNA remodeling (bending and wrapping), UTP-dependent reiterative transcription initiation, and stringent control by the alarmone ppGpp. At the enzyme level, CPSase activity is tightly controlled by allosteric effectors originating from different pathways: an inhibitor (UMP) and two activators (ornithine and IMP) that antagonize the inhibitory effect of UMP. Furthermore, it is worth noticing that all reaction intermediates in the production of CP are extremely reactive and unstable, and protected by tunneling through a 96 Å long internal channel.

## CP at the crossroad of arginine and pyrimidine synthesis

**Fig. 1:**
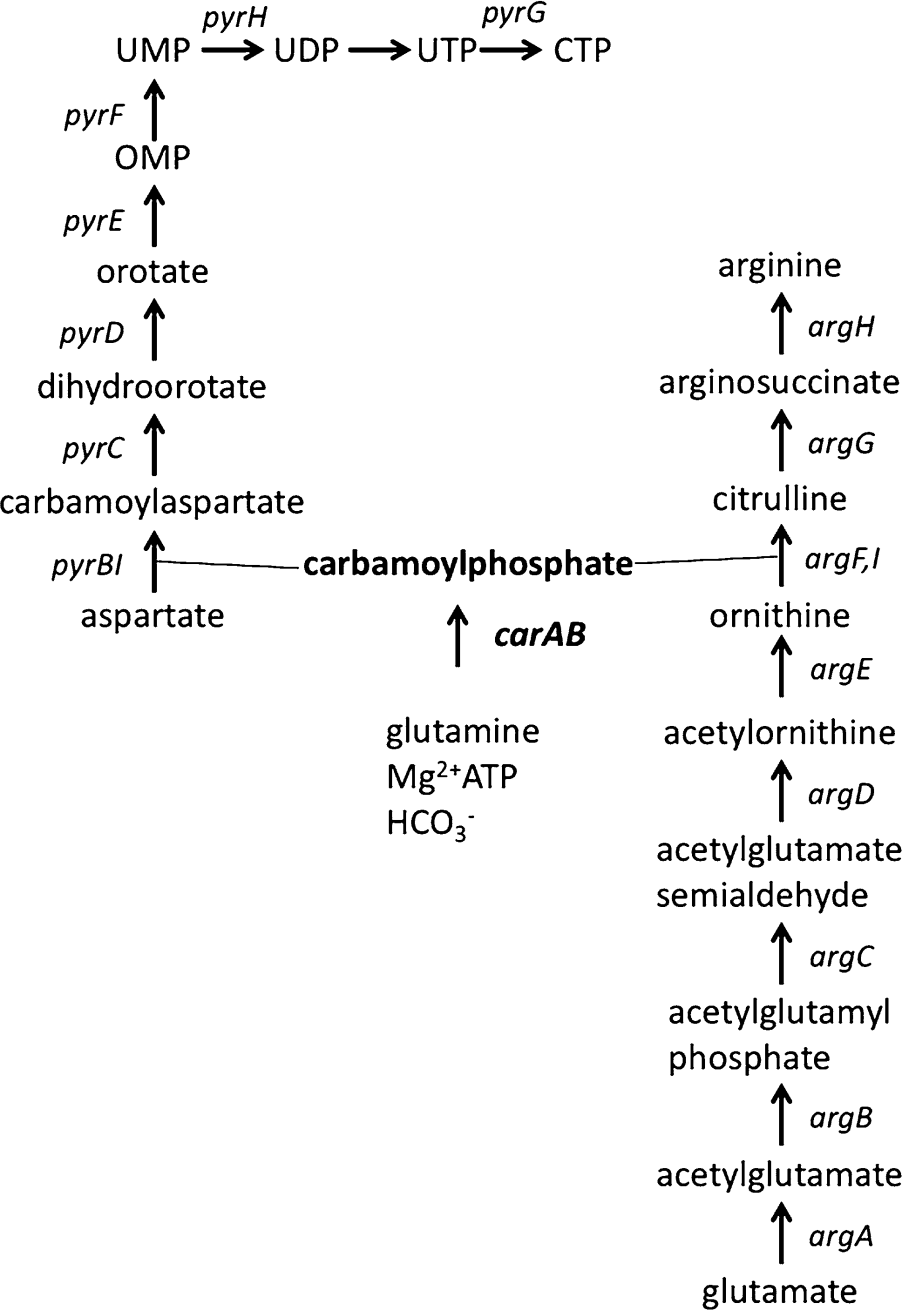
Biosynthesis of arginine and pyrimidines. The names of structural genes encoding the enzymes catalyzing the different steps of de novo arginine and pyrimidine synthesis are indicated as follows: *argA* (*N*-acetylglutamate synthase), *argB* (*N*-acetylglutamate kinase), *argC* (*N*-acetylglutamylphosphate reductase), *argD* (*N*-acetylornithine transaminase), *argE* (*N*-acetylornithinase), *argF* and *argI* (ornithine transcarbamylase, two isoenzymes), *argG* (argininosuccinate synthetase), *argH* (argininosuccinase), *carAB* (carbamoylphosphate synthase), *pyrBI* (aspartate transcarbamylase with *pyrB* encoded catalytic and *pyrI* encoded regulatory subunit), *pyrC* (dihydroorotase), *pyrD* (dihydroorotate dehydrogenase), *pyrE* (orotate phophoribosyltransferase), *pyrF* (orotidine-5′-phosphate decarboxylase), *pyrH* (UMP kinase), *pyrG* (CTP-synthase)

Carbamoylphosphate (CP) plays a dual metabolic role as it is required for both the de novo synthesis of arginine and pyrimidines (Fig. 1). The existence of a precursor common to both pathways was suggested by the isolation of one-step double auxotrophic *E. coli* mutants by RR Roepke, as quoted in (Tatum 1946). Ten years later, after extensive biochemical work, the molecule was identified as CP (Jones et al. 1955). In *Escherichia coli*, *Salmonella typhimurium* and related organisms, CP is synthesized from glutamine, two molecules of Mg^2+^ATP and bicarbonate by a single enzyme, carbamoylphosphate synthase (E.C. 6.3.5.5), encoded by the *carAB* operon (or its equivalent *pyrA* in *Salmonella*) (Piérard and Wiame 1964; Piérard et al. 1965; Abd-el-Al and Ingraham 1969; Abdelal and Ingraham 1975). This particular situation generates the need for a complex and tight control of CP production and its utilization for the de novo synthesis of arginine and pyrimidines in function of the cellular needs. In the arginine biosynthetic pathway, CP and ornithine are the substrates of ornithine carbamoyltransferase (OTCase) for the production of citrulline, which is further converted to arginine in two enzymatic steps (reviewed in Charlier and Glansdorff 2004). In the first committed step of pyrimidine biosynthesis, CP and aspartate are condensed into carbamoylaspartate, a reaction catalyzed by aspartate transcarbamylase (ATCase) (Lipscomb and Kantrowitz 2012), a paralog of OTCase (Labedan et al. 1999). A similar situation prevails in *Salmonella typhimurium* (Abdelal and Ingraham 1975), *Serratia marcescens* (Crane and Abdelal 1980), *Pseudomonas aeruginosa* (Abdelal et al. 1983), *Proteus mirabilis* (Prozesky and Coetzee 1966), *Neisseria gonorrhoeae* (Shinners and Catlin 1982) and enteric bacteria in general (Cunin et al. 1986). However, other microorganisms such as the Gram-positive bacteria *Bacillus subtilis* (Paulus and Switzer 1979), *Geobacillus stearothermophilus* (Yang et al. 1997) and *Lactobacillus plantarum* (Nicoloff et al. 2000), the yeast *Saccharomyces cerevisiae* and fungi such as *Neurospora crassa* (Lacroute et al. 1965; Bernhardt and Davis 1972; Davis 1986) possess two specialized and distinctly regulated CPSases, one for each pathway. In *S. cerevisiae* and *N. crassa*, the pyrimidine-specific CPSase encoded by the URA2 gene is part of a multifunctional protein exhibiting CPSase and ATCase activity (Lue and Kaplan 1969; Davis and Woodward 1962; Finck et al. 1965). In addition, the bifunctional protein carries an inactive dihydroorotase (DHO)-like domain in the linker connecting the CPSase and ATCase domains (Souciet et al. 1982, 1989). In the multifunctional CAD (CPSase, ATCase, DHOase) protein of higher eukaryotes, the dihydroorotase domain is functional. In some organisms, including bacteria such as *Pseudomonas aeruginosa*, *Bacillus licheniformis*, *Streptococcus faecalis* and *Lactobacillus* species, but also protists, moulds and halophilic archaea, CP can also be produced from citrulline by a catabolic OTCase of the arginine deiminase pathway, in which CP is then further used to generate ATP and ammonia (reviewed in Leroy and Charlier 2017). In hyperthermophilic archaea such as *Pyrococcus* species, a carbamate kinase rather than a CPSase appears to be responsible for CP synthesis (Purcarea et al. 1996; Durbecq et al. 1997; Rámon-Maiques et al. 2000; Uriarte et al. 1999; Marina et al. 1999; Alcántara et al. 2000). For additional information on CP producing and consuming enzymes in various organisms, including humans, the reader is referred to a recent review by Shi et al. (2018).

## Transcriptional regulation of the *E. coli carAB* operon

### Tandem promoters direct transcription of the *carAB* operon

**Fig. 2:**
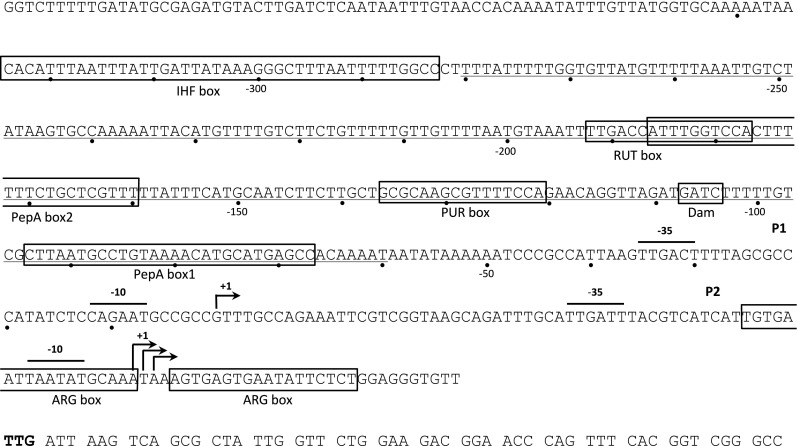
Sequence and outline of the *E. coli carAB* control region. P1 and P2 represent the tandem pair of promoters directing *carAB* transcription, with indication of their respective − 10 and − 35 promoter elements, and transcription initiation site(s) (arrows). Distances are indicated with respect to the start of P1 transcription. Boxed sequences represent binding sites for the various transcription factors and trigger enzymes modulating transcription initiation at the P1 and P2 promoters. The two ARG boxes constitute the binding site for hexameric arginine-bound ArgR. The PUR box is the target of dimeric guanine or hypoxanthine-bound PurR. The RUT box is the target of unliganded dimeric RutR. The IHF box is the binding site of heterodimeric integration host factor (IHF), a nucleoid-associated protein. PepA1 and PepA2 represent sites of tight contact with the hexameric trigger enzyme PepA as identified in DNase I footprinting. The approximately 230 bp long region that gets wrapped around PepA is underlined. Dam represents the GATC sequence that is methylated by deoxyadenosine methyltransferase, a reaction that is inhibited upon binding of PepA and/or IHF. Notice that the initiation codon for *carA* mRNA translation is TTG (in bold)

The synthesis of *E. coli* CPSase is predominantly regulated at the transcriptional level as indicated by an almost perfect correlation between the levels of mRNA and enzyme activity over a large repression–derepression range (Piérard et al. 1980). The small glutaminase and large synthase subunit of the heterodimeric enzyme are encoded by the *carA* and *carB* gene, respectively, which from an operon (Mergeay et al. 1974; Gigot et al. 1980; Crabeel et al. 1980). It was first observed that the synthesis of CPSase is partially repressed upon growth in the presence of an excess of either arginine or a pyrimidine (usually uracil), and nearly completely in the presence of both, a phenomenon that was described as “cumulative repression” (Piérard and Wiame 1964; Piérard et al. 1965). Later on, it was demonstrated that the operon is transcribed from two adjacent promoters (Fig. 2), with startpoints 67 nt apart, and that excess pyrimidines switches off the upstream promoter P1, whereas excess arginine represses the downstream promoter P2 but does not affect initiation at P1 (Piette et al. 1984; Bouvier et al. 1984). In the genomes of *E. coli* and *S. typhimurium*, the operon is not directly linked to any other arginine or pyrimidine gene or transcriptional regulator affecting its expression.

## Regulation of P2 promoter activity by arginine-bound ArgR

In vivo and in vitro P2 is repressed (about 50-fold as measured in a P2 only fusion construct) in the presence of excess arginine by hexameric ArgR (Piérard et al. 1972; Lissens et al. 1980; Charlier et al. 1988, 1992). ArgR is the repressor of arginine biosynthesis in *E. coli* and belongs to the winged helix-turn-helix (wHTH) family of DNA-binding transcription factors (Lim et al. 1987; Maas 1994; Van Duyne et al. 1996; Sunnerhagen et al. 1997). A single hexameric arginine-bound ArgR molecule cooperatively binds to two 18 bp long imperfect palindromes (ARG boxes) separated by three bp that partially overlap the − 35 promoter element (Fig. 2) and contacts major and minor groove determinants of the P2 operator, all aligned on one face of the DNA helix (Charlier et al. 1992; Wang et al. 1998). Mutations altering the sequence of these ARG boxes alleviate repression of the *carAB* operon by arginine and reduce the in vitro binding affinity for ArgR (Roovers et al. 1988; Charlier et al. 1992; Wang et al. 1998). Binding of RNA polymerase (RNAP) and ArgR to the P2 promoter region was shown to be mutually exclusive (Charlier et al. 1988). In contrast, ArgR does not impede the binding of RNAP to the P1 promoter and transcription initiated from this upstream promoter can proceed even in the presence of excess arginine (Piette et al. 1984; Charlier et al. 1988). Interestingly, ArgR-mediated repression of P2 was found to be stronger upon silencing initiation at P1 (either by physiological repression or mutational inactivation), suggesting that the ArgR•P2 operator complex is destabilized by RNAP binding at P1 or transcription initiated from P1 (Charlier et al. 1988). A very similar situation prevails in *S. typhimurium*, where *carAB* transcription is equally initiated from two tandem promoters and P2 repressed by ArgR binding to a pair of adjacent ARG boxes (Kilstrup et al. 1988; Lu et al. 1992). Interestingly, attenuation control that is operative in the control of many amino acid biosynthetic pathways is not involved in the regulation of P2 activity and neither in regulation of any other gene or operon of arginine biosynthesis in *E. coli* (Cunin et al. 1983).

## Regulation of transcription initiation at P1

In contrast to the rather simple control mechanism operating at P2, unraveling the regulatory mechanisms modulating P1 activity turned out to be particularly challenging, and in spite of the identification of numerous players, the picture is still incomplete. It was clearly established that excess pyrimidines switches off P1 transcription (Charlier et al. 1988), but a general regulator of pyrimidine biosynthesis does not exist in *E. coli*, where all genes of pyrimidine biosynthesis are non-coordinately regulated by a variety of mechanisms (reviewed in Turnbough and Switzer 2008). In contrast, numerous DNA-binding transcription factors, nucleoid-associated factors (NAPs) and trigger enzymes (enzymes with a second unlinked gene regulatory function) were found to interact with an approximately 350 bp region upstream of the P1 promoter (Fig. 2). Roovers et al. (1988) isolated a first *trans*-acting regulatory element, CarP, in a search for P1 derepressed mutants. In *carP* mutants, P1 activity is no longer repressed upon uracil supplementation of the medium and about twofold higher than in the isogenic wild-type strain grown on minimal medium (Roovers et al. 1988; Charlier et al. 2000). Surprisingly, CarP turned out to be identical to PepA (Charlier et al. 1995b), an important Mn^2+^-dependent aminopeptidase in *E. coli* (Vogt 1970), and XerB, an auxiliary protein involved in conjunction with ArgR (alias XerA) in site-specific resolution of ColE1 multimers into the monomeric constituents (Guathakurta and Summers 1995; Stirling et al. 1989; Alén et al. 1997). Subsequent work indicated that the catalytic activity of PepA in not required for transcriptional regulation and that the protein binds to the P1 control region of *E. coli* and *S. typhimurium* in vitro in the absence of any effector molecule, and in vivo affects the methylation status of a GATC site in the P1 control region (Charlier et al. 1995a, b, 2000). PepA is a 330 kDa large homohexameric molecule (dimer of trimers) of which the structure has been solved at 2.5 Å resolution (Sträter et al. 1999). Each subunit consists of two domains: a smaller N-terminal domain (residues 1–166) is connected via a 26 amino acid long α-helix to the large C-terminal domain (residues 193–503) that bears the catalytic site. PepA does not possess a canonical DNA-binding motif, but on the basis of structural data and mutant studies, the N-terminal domain was shown to play an important role in DNA binding. DNA is proposed to dock into a groove running on the surface of the C-terminal domain of the protein and is bound by patches of basic residues that constitute the major N-terminal DNA-binding determinants at the ends of the groove (Sträter et al. 1999; Charlier et al. 2000; Reijns et al. 2005). DNase I footprinting assays and atomic force microscopy of PepA•P1 operator DNA complexes of *E. coli* and *S. typhimurium* indicated the deformation of an ~ 230 bp large region, extending from position − 280 to − 60 with respect to the start of P1 transcription, by wrapping around a single hexameric PepA molecule (Fig. 2) (Charlier et al. 1995b; Minh et al. 2009). This region comprises the binding sites for two transcriptional regulators, RutR and PurR, which equally interact with the P1 operator (see below). DNA topology assays indicated that wrapping of the P1 operator DNA around hexameric PepA generates a positive toroidal supercoil (Nguyen Le Minh et al. 2016). In view of the ligand-independent DNA binding of PepA, its multifunctional character, and the structural deformations, PepA was originally supposed to play a merely architectural role in P1 regulation. However, recently single-round in vitro transcription assays clearly demonstrated that PepA is a repressor in its own right, and hence a trigger enzyme, that specifically inhibits transcription initiation at P1, and the associated topological change is an integral part of the regulatory process (Nguyen Le Minh et al. 2016).

Besides PepA, three other proteins directly bind to the P1 control region and participate in modulation of P1 activity: RutR, the master regulator of genes involved in pyrimidine degradation (Shimada et al. 2007), PurR, the regulator of purine metabolism (Rolfes and Zalkin 1988; Cho et al. 2011), and the nucleoid-associated and DNA-bending protein IHF (integration host factor). RutR is a member of the TetR family of allosteric transcriptional regulators and was originally identified as the transcriptional regulator of the *rutABCDEFG* operon encoding a novel pathway for pyrimidine utilization in *E. coli* (Loh et al. 2006). Subsequent genomic SELEX and ChIP chip experiments identified 20 RutR binding sites on the *E. coli* genome, among which the *carAB* control region exhibits the highest affinity for the regulator (Shimada et al. 2008). RutR is an activator of transcription initiation at P1 and this stimulatory effect is strongly reduced in uracil-supplemented medium (Shimada et al. 2007). DNase I footprinting and high-resolution contact mapping of RutR•DNA complexes identified a 30 bp region of interaction, far upstream of the P1 promoter (from -200 to -170; Fig. 2) that bears a RUT box (-177 to -192) (Shimada et al. 2007; Nguyen Ple et al. 2010). In contrast to previous work (Shimada et al. 2007), structural work, modeling and mutant studies established that uracil but not thymine is the physiologically relevant ligand that abolishes DNA binding (Nguyen Le Minh et al. 2015). As the PepA and RutR binding sites overlap, competition in the binding of PepA and RutR has been proposed but not proven yet (Shimada et al. 2007).

In both *E. coli* and *S. typhimurium* P1 is about three to fourfold down-regulated by excess purines. This effect was first observed in *S. typhimurium* (Lu et al. 1995) and later in *E. coli*, where the molecular mechanism was studied (Devroede et al. 2004). PurR-mediated repression of P1 activity is much lower than purine-dependent repression of purine biosynthetic genes, but comparable to PurR-mediated repression of several other genes, including *pyrC* (DHOase) and *pyrD* (DHODase, dihydroorotate dehydrogenase) of pyrimidine biosynthesis (Choi and Zalkin 1990; Wilson and Turnbough 1990). In the presence of either guanine or hypoxanthine, the physiological effectors of PurR, the regulator binds to a 16 bp PUR box centered around position − 128.5 upstream of the start of P1 transcription (Fig. 2) and bends the operator by ~ 97° (Devroede et al. 2004), comparable to the PurR-induced deformation of the *purF* operator (Schumacher et al. 1997). This upstream position of the PurR-binding site in the P1 operator is unusual in the PurR regulon, where the binding site mostly partially overlaps the promoter or exceptionally works as a roadblock from a promoter downstream binding site (He and Zalkin 1992). In vitro transcription assays with purified PurR and analyses of the purine/PurR effect in various *cis*- and *trans*-acting mutants indicated that liganded PurR is by itself unable to repress P1 activity (Devroede et al. 2004, 2006). Instead, PurR appears to rely on the PepA-induced remodeling of the P1 control region to exert its regulatory effect. As a consequence, PurR- and PepA-mediated repression of P1 are structurally and functionally coupled, which is unprecedented in the action of PurR.

Integration host factor (IHF), a heterodimeric protein of similar subunits encoded by the *himA* and *hip* (alias *himD)* genes, and first discovered as a host-encoded protein involved in site-specific integration of bacteriophage lambda (Miller et al. 1979) is a multifunctional DNA-bending NAP that shows some sequence specificity for DNA binding (Goodrich et al. 1990; Goosen and van de Putte 1995). IHF was shown to bind to a highly A + T rich stretch far upstream (− 324 to − 287) of the start of P1 transcription in *E. coli* and *S. typhimurium* (Charlier et al. 1993). It is the most upstream located binding site in the P1 operator yet identified (Fig. 2). IHF stimulates P1 activity in cells grown in minimal medium but potentiates the pyrimidine-specific repression in uracil-supplemented medium. IHF thus appears to exert antagonistic effects and is required for both maximal expression and full repression of P1 (Charlier et al. 1993). As also observed for PepA, binding of this abundant NAP affects the methylation status of a GATC site 106 bp upstream of the start of P1 transcription (Charlier et al. 1994, 1995a). However, inhibition of methylation of this site appears to be a mere passive consequence of protein binding and not an active component of the regulatory process, since mutants of the GATC site that cannot be methylated are not impaired in P1 regulation (Charlier et al. 1995b).

Fis (factor for inversion stimulation), another NAP, was equally proposed to be involved in regulation of *carA* expression (Bradley et al. 2007). Fis is the most abundant NAP during early exponential phase (> 50,000 molecules/cell), when *E. coli* cells are dividing rapidly, but its concentration decreases tremendously during stationary phase (Ali Azam et al. 1999). Fis is involved in nucleoid structuring through binding to A-/AT tracts, but its local positive or negative effects on gene regulation are not well-understood (Cho et al. 2008). DNA microarray analyses in wild-type and mutant *fis E. coli* in different growth stages indicated an approximately twofold regulation of *carA* expression by Fis and predicted the presence of four A + T rich binding sites for Fis in the *carAB* control region, of which three are upstream of P1 and one overlapping the P2 promoter (Bradley et al. 2007). However, even though there is in vitro binding of purified Fis protein to P1 and P2 operator fragments observed in electrophoretic mobility shift assays (EMSA) in our laboratory, these complexes appeared to be non-specific. Furthermore, comparative reporter gene expression studies performed with isogenic wild-type and *fis* deletion strains in exponential growth phase did not reveal significant differences in P1 and P2 promoter activity (Islam Emdadul 2011).

Finally, Kholti et al. (1998) gathered evidence indicating that PyrH (UMP kinase) directly participates in pyrimidine-specific modulation of P1 activity. Interestingly, *E. coli* UMP kinase is a homohexameric protein, whereas most other AMP, GMP and UMP kinases are small monomeric proteins that share significant sequence homology (Briozzo et al. 2005). Furthermore, the enzyme does not display significant sequence homology with known UMP kinases of eukaryotic origin (Liljelund et al. 1989; Wiesmüller et al. 1990). In *E. coli, pyrH* is an essential gene and UMP kinase a highly regulated enzyme (inhibited by UTP, activated by GTP) that controls the de novo synthesis of all other pyrimidine nucleotides (Serina et al. 1995) (Fig. 1). In the search of P1 derepressed mutants on uracil-supplemented medium, *pyrH* mutants bearing a single amino acid substitution and retaining a quasi-normal UMP kinase activity were found to be impaired in P1 regulation (Kholti et al. 1998). Overexpression of the UMP–CMP kinase gene of *Dictyostelium discoideum* in such a mutant resulted in elevated UMP kinase levels, but did not restore normal control of P1, indicating that the slight reduction in UMP kinase activity and the potential concomitant reduction in the pyrimidine nucleotide pool is not responsible for the regulatory deficiency (Kholti et al. 1998). In vitro DNA-binding assays (EMSA and DNase I footprinting) with purified PyrH in the presence/absence of potential ligands did not reveal direct binding of the trigger enzyme to the P1 control region. Rather, on basis of preliminary yeast two hybrid assays (Nguyen 1998) and the opposite charge distribution on the surface of PyrH and PepA, two hexameric trigger enzymes involved in regulation of P1 activity, PyrH was proposed to be attracted to the P1 control region by protein–protein interaction (Briozzo et al. 2005; Marco-Marín et al. 2005), but this hypothesis has not been further validated yet.

Besides and independent of the complex TF-dependent regulation of transcription initiation that is operative at rather high UTP levels (0.9–1.4 mM), P1 activity is also regulated by a reiterative transcription control mechanism when the UTP level is low (0.9–50 µM) (Han and Turnbough 1998). These very low intracellular levels of UTP are not characteristic of exponentially growing prototrophs, but may occur subsequent to a sudden shift from high to low pyrimidine-supplemented medium, and in pyrimidine auxotrophs grown under limiting pyrimidine supply. Reiterative transcription, also known as transcriptional slippage or RNAP stuttering, is the repetitive addition of the same nucleotide to the 3’-end of the nascent transcript and results from the slippage between the transcript and the template DNA in a homopolymeric sequence. The phenomenon has been observed with RNAPs from all domains of life and with viral enzymes as well (Jacques and Kolakofsky 1991; Xiong and Reznikoff 1993; Cheng et al. 2001). In the context of pyrimidine metabolism, UTP-dependent reiterative transcription was first discovered for the *pyrBI*, *codBA* and *upp* genes and operons of *E. coli* (Liu and Turnbough 1989; Qi and Turnbough 1995; Tu and Turnbough 1997) and later for the *carAB* operon, where the effect is less pronounced (approximately threefold and two to threefold lower than for *pyrBI*) (Han and Turnbough 1998). P1 transcription starts with a G-residue followed by a series of three U residues (5′-GUUUGC-3′) (Fig. 2). Weak base pairing between the nascent GUUU transcript and its complementary DNA template allows for reversible one-base slippage and in the presence of high UTP concentrations, an extra U residue may be added to the 3′-end. Repeated rounds of slippage and extension may result in transcripts bearing long runs of U residues (5′-GUUUUn, with *n* = 1 to > 30), which are not further elongated into productive full-length mRNA molecules (Han and Turnbough 1998). At low UTP levels, the probability of inserting an extra U residue is much lower and even if slippage occurs, correct repositioning of the GUUU transcript will allow normal template-dependent transcription elongation. The importance of the polymeric stretch in the process is underscored by the observation that single T to G or T to C substitutions that interrupt the run of three T residues in the non-template strand of the initially transcribed region of the P1 promoter abolish the reiterative transcription control (Han and Turnbough 1998). Furthermore, mutant studies performed with the P1 promoter and comparative studies with the *pyrBI* and *galETKM* operons revealed the importance for stuttering of the initiating nucleotide (with A resulting in more reiterative transcription than G) and of the spacing between the − 10 promoter region and the transcription start site (with an 8 bp spacer generating more stuttering than the more canonical 7 bp linker) (Han and Turnbough 2014).

Finally, the P1 promoter was also shown to be subject to stringent control by the alarmone ppGpp (Bouvier et al. 1984). In a *relA*^+^ strain induction of isoleucine starvation by addition of valine to the culture medium resulted in an important increase in the ppGpp concentration and a concomitant approximately twofold reduction of P1 activity, which is very similar to the effect of stringent control on expression of the *pyrBI* operon (Turnbough 1983). However, the latter was measured in vitro in the absence of the small protein DksA, which is known to enhance the effect of ppGpp. The in vivo effect might thus be even stronger. It is worth noticing that in agreement with many other stringent promoters, P1 exhibits a six bp G + C rich sequence stretch (GCCGCC) immediately following the Pribnow box and preceding the G start nucleotide of the P1 mRNA (Fig. 2) that might function as a discriminator box and high-energy barrier for the isomerization from the closed to the open promoter complex (Dalebroux and Swanson 2012).

## *E. coli* CPSase: reaction intermediates and enzyme structure

**Fig. 3:**
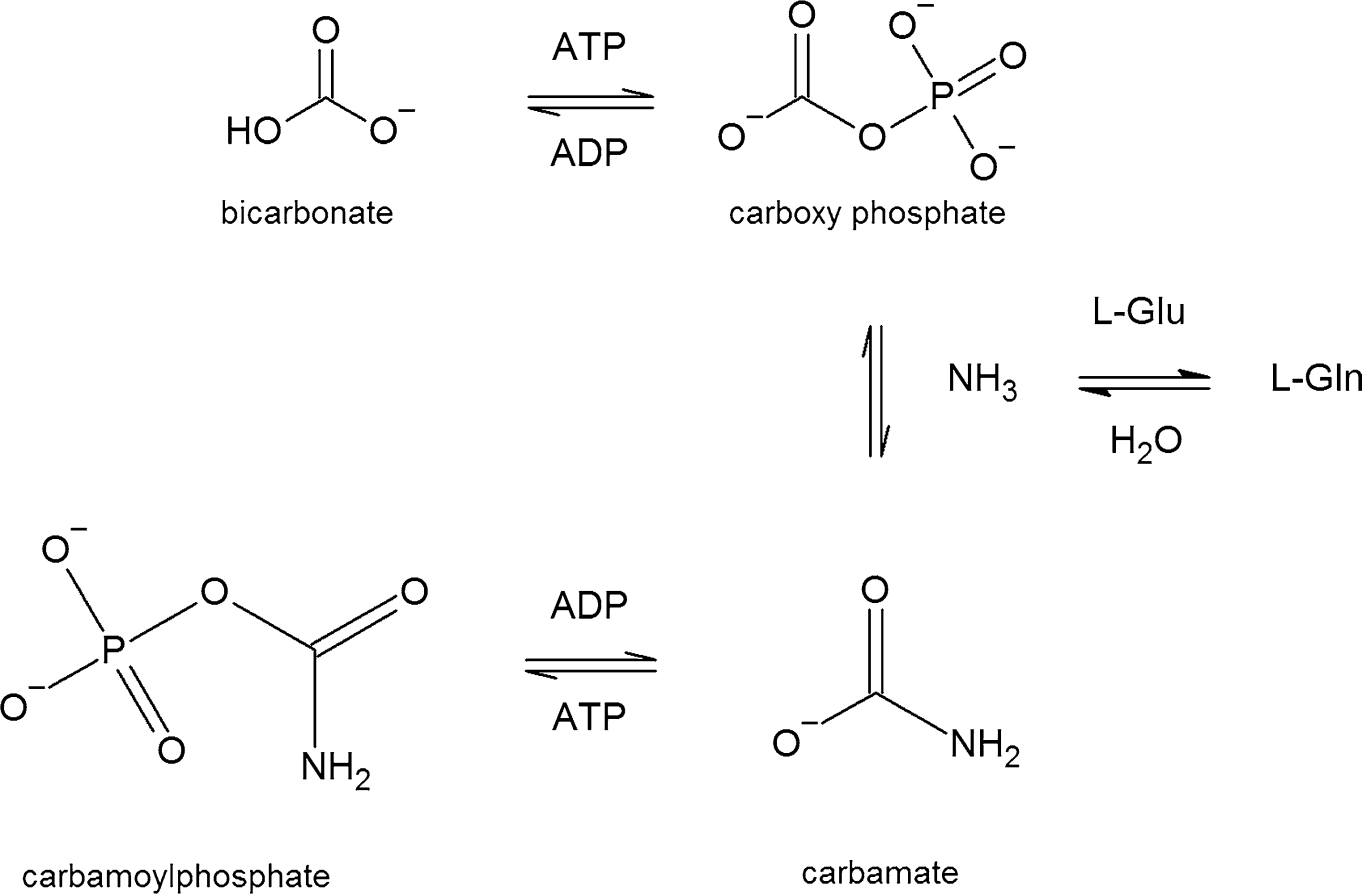
Reaction scheme of the synthesis of carbamoylphosphate by *E. coli* CPSase using glutamine as nitrogen donor

All CPSases use one molecule of bicarbonate, two molecules of Mg^2+^ATP, and one molecule of either glutamine or ammonia to synthesize CP (Fig. 3) (Jones and Lipman 1960; Anderson and Meister 1965; Shi et al. 2018). The preferred and physiologically relevant nitrogen donor for *E. coli* CPSase, and by extension for all bacterial CPSases, is glutamine (Piérard and Wiame 1964; Cunin et al. 1986; Meister 1989) (Fig. 3). Sequence conservation and structural characteristics indicate that the enzyme is part of the class I amidotransferase family of enzymes (Raushel et al. 1999). The reaction with ammonia is possible but requires very high and likely physiologically irrelevant concentrations (Rubino et al. 1987).

**Fig. 4:**
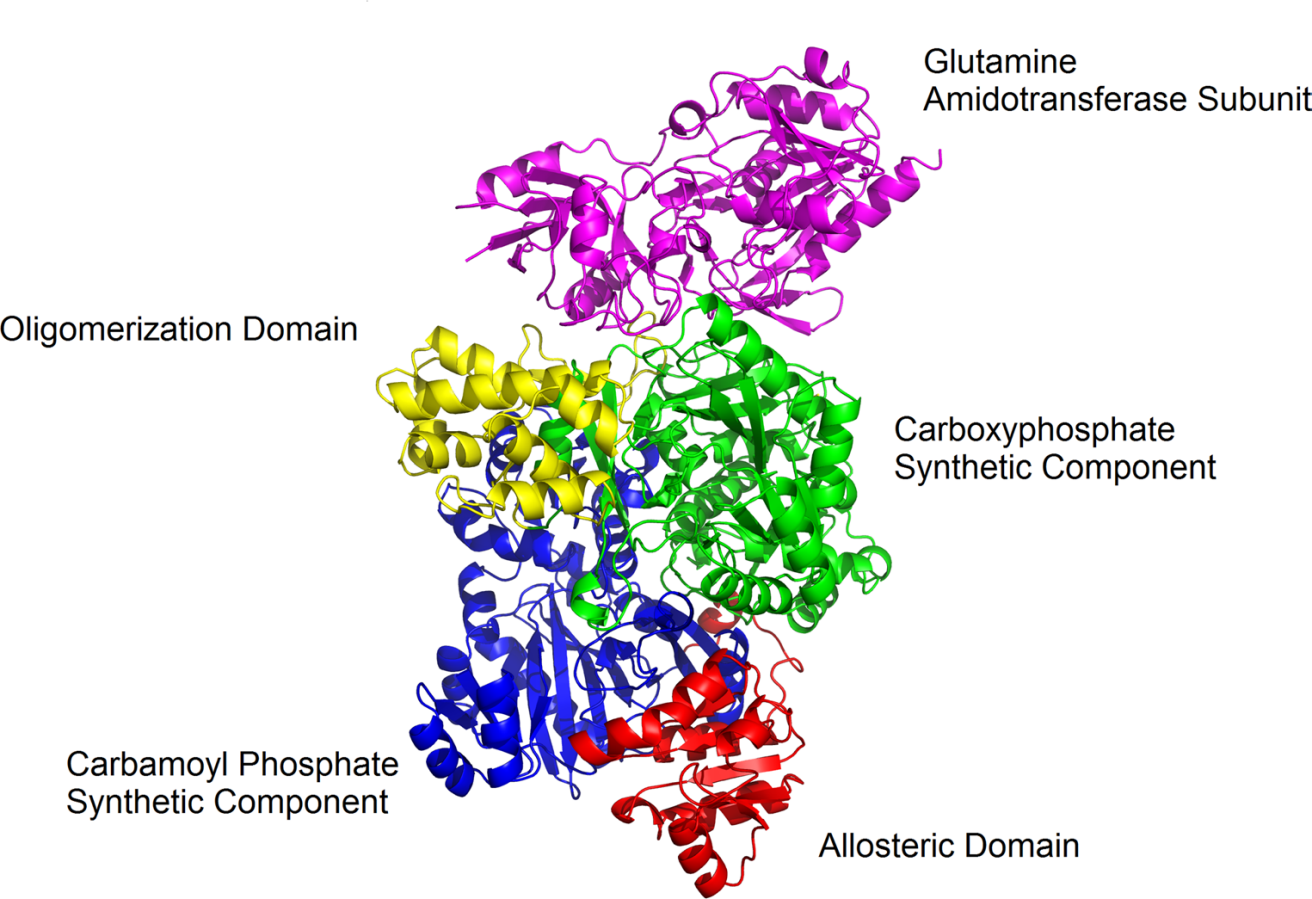
Ribbon presentation of the structure of heterodimeric (αβ) *E*. *coli* carbamoylphosphate synthase (pdb-1JDB) [133]. The small, *carA* encoded, glutamine amidotransferase subunit (magenta), and each domain of the large, *carB* encoded, catalytic subunit are indicated in different colors: carboxyphosphate synthetic component (green), oligomerization domain (yellow), carbamoylphosphate synthetic component (blue), allosteric domain (red). The figure was prepared with PyMOL

*E. coli* CPSase consists of two subunits, a small glutaminase subunit of ~ 41,000 (382 aa residues) encoded by *carA* and a large *carB* encoded subunit of 118,000 (1073 aa residues), that catalyzes the synthesis of CP from ammonia and also carries all the effector binding sites (Trotta et al. 1971; Matthews and Anderson 1972; Trotta et al. 1974a, b) (Fig. 4). *carB* is clearly the result of a gene duplication event, which suggests that CPSase evolved from a more primitive enzyme exhibiting kinase activity (Nyunoya and Lusty 1983; Lawson et al. 1996). The two halves of this large subunit exhibit 39% amino acid sequence identity, are independently folded, and functionally equivalent (except for the regulatory effects) whereby each part fixes one of the two Mg^2+^ATP molecules required for the synthesis of one molecule of CP (Guy and Evans 1996; Guy et al. 1997; Stapleton et al. 1996; Javid-Majd et al. 1996; Thoden et al. 1997). Combined structural, biochemical and genetic work provided detailed insight into the organization of the different domains of the enzyme (Fig. 4). The large subunit of *E. coli* CPSase is composed of four major domains (Thoden et al. 1997, 1999b). The carboxyphosphate domain (residues 1–400) and the homologous carbamate phosphorylation domain (residues 553–933; carbamoyl phosphate synthetic unit) exhibit nearly the same overall tertiary fold and are related by an axis of twofold rotational symmetry. Remarkably, the active sites of these two phosphorylation domains are separated by nearly 40 Å. These two domains are connected by an additional domain (residues 401–552) that appears to be required for oligomerization of the enzyme. Finally, the very C-terminus (residues 933–1073) constitutes the allosteric domain to which the allosteric effectors of CPSase activity bind (Rubio et al. 1991; Cervera et al. 1996; Czerwinsky et al. 1995; Delannay et al. 1999; Holden et al. 1999; Thoden et al. 1999c, d). The small glutaminase subunit makes contact with the carboxyphosphate domain, to which ammonia is delivered, and the domain involved in oligomerization. Cys-269 and His-353 of the glutaminase subunit are two residues essential for the hydrolysis of glutamine that passes through a thioester intermediate (Miran et al. 1991; Khedouri et al. 1966; Pinkus and Meister 1972; Anderson and Carlson 1975; Rubino et al. 1986; Mullins et al. 1991; Thoden et al. 1998, 1999a; Huang and Raushel 1999; Rishavy et al. 2000). From the structure, it appears that the three active sites in the enzyme are located far apart but connected by an intramolecular tunnel of 96 Å that leads from the glutamine binding site on the small subunit over the carboxyphosphate site (about 45 Å away) all the way to the carbamate phosphorylation site (another ~ 40 Å long tunnel) (Thoden et al. 1997; Huang and Raushel 2000a, b; Huang et al. 2001; Kim et al. 2002; Kim and Raushel 2004a, b; Fan et al. 2008, 2009; Lund et al. 2010). It is proposed that tunneling of the highly unstable reaction intermediates (Fig. 3) through the interior of the enzyme plays a crucial role in their protection (Thoden et al. 1997; Huang et al. 2001). CP itself is also a very thermolabile (half-life of < 2 s at 100 °C) and potentially harmful metabolite, as its decomposition produces the highly toxic cyanate (Allen and Jones 1964; Wang et al. 2008). However, binding of CP as a substrate to the active site of the tributary OTCase and ATCase enzymes strongly reduces its rate of thermal decomposition (Wang et al. 2008). In *E. coli*, there are at present no direct indications of further protection of CP through channeling between CPSase and the major CP consuming enzymes. This is, however, different in thermophilic organisms, where thermal degradation of CP would appear even more problematic. In the hyperthermophilic archaea *Pyrococcus furiosus* and *P. abyssi*, growing optimally at 100 °C, there is evidence for a weak physical association between the carbamate kinase-like CPSase and either OTCase or ATCase into a multienzyme cluster in which CP is directly transferred from the site of its synthesis to the active site of the consuming enzymes (Legrain et al. 1995; Purcarea et al. 1999; Massant et al. 2002; Massant and Glansdorff 2004, 2005). Channeling of CP to ATCase has also been demonstrated in the hyperthermophilic bacterium *Aquifex aeolicus* (Purcarea et al. 2003). Finally, partial channeling of CP in mesophilic hosts has also been shown to occur within the multienzymatic pyrimidine-specific CPSase–ATCase enzyme from the lower eukaryotes *S. cerevisiae* and *N. crassa*, and the CAD enzyme of mammals (Belkaïd et al. 1988; Penverne et al. 1994; Williams et al. 1970, 1971; Christopherson and Jones 1980; Irvine et al. 1997; Serre et al. 1999).

## Allosteric regulation of CPSase activity

*E. coli* CPSase is a key enzyme involved in the synthesis of pyrimidine nucleotides and arginine, and hence occupies a strategic position in the production of building blocks for the synthesis of nucleic acids and proteins. This unique situation is not only reflected in the transcriptional control of the *carAB* operon as described above, but equally in the control of the enzyme’s activity. *E. coli* CPSase is an allosteric enzyme that is activated by ornithine and IMP, and inhibited by UMP (Piérard 1966; Anderson and Marvin 1968; Trotta et al. 1974a). The activating role of ornithine is crucial in view of the implication of the unique CPSase in arginine biosynthesis (Fig. 1). In presence of arginine, the synthesis of N-acetylglutamate synthetase, the first enzyme of arginine synthesis starting from glutamate, is repressed by arginine-bound ArgR and its activity feedback inhibited by arginine (Charlier and Glansdorff 2004). As a consequence, all pathway intermediates are present in limiting concentrations in this situation. When the arginine pool becomes too low to ensure protein synthesis, both repression and feedback inhibition of *N*-acetylglutamate synthetase are lifted and the concentration of ornithine increases. However, further conversion of the latter into citrulline requires CP. Activation of CPSase by accumulating ornithine (and concomitant derepression of the P2 promoter) thus ensures sufficient CP supply for the synthesis of arginine in this condition. The antagonistic effects of UMP and IMP may contribute to a correct balance between the production of purine and pyrimidine nucleotides in the cell. All three effector molecules primarily exert their effect on CPSase activity by modifying the apparent affinity of the enzyme for Mg^2+^ATP by approximately one order of magnitude, hence modulating the saturation of the enzyme by its substrate (Piérard 1966; Anderson and Meister 1966; Braxton et al. 1992, 1996).

The structure of *E. coli* CPSase was determined in the presence of different substrates and effectors, hence the binding sites for ornithine, IMP and UMP could be located and they all bind the C-terminal allosteric domain of CPSase (Thoden et al. 1997, 1999b, c, d, 2004). Previous studies performed with analogs of UMP and IMP already indicated that these antagonistic nucleotide effectors may bind the same or overlapping sites, but that this site is distinct from the ornithine binding site (Boettcher and Meister 1981, 1982; Mora et al. 1999; Fresquet et al. 2000; Braxton et al. 1999). The binding of UMP and ornithine essentially affect the binding of the second Mg^2+^ATP molecule (Braxton et al. 1992, 1999). Furthermore, the binding of ornithine and IMP decreases the affinity of the enzyme for the inhibitor UMP and vice versa, the binding of UMP lowers its affinity for the activators (Anderson 1977; Robin et al. 1989; Rubio et al. 1991). However, the activation by ornithine completely dominates the effects of the nucleotides (Braxton et al. 1999; Delannay et al. 1999). The inhibition in the binding of IMP by UMP is due to a competition in the binding of these two nucleotides for a common binding site (Anderson 1977; Boettcher and Meister 1981, 1982; Braxton et al. 1999; Bueso et al. 1999). From the solution of a co-crystal structure at 2.1 Å resolution IMP is now known to bind at the C-terminal portion of a five-stranded parallel β-sheet formed by the residues Ser937 to Lys1073 (Thoden et al. 1999c). Mutant studies and photolabeling assays indicated that Thr977 and Lys993 are crucial residues for UMP-dependent inhibition and UMP binding, respectively (Czerwinsky et al. 1995; Cervera et al. 1996; Pierrat and Raushel 2002). Structural data indicate that ornithine binds at the interface between the allosteric and the carbamoylphosphate domains. The carboxylate group of ornithine lies within hydrogen-bonding distance to both the backbone amide group and the side chain hydroxyl group of Thr1042 (Thoden et al. 1997, 1999b). Sequence determination and biochemical characterization of *carB* mutants isolated by Mergeay et al. (1974) pinpointed the crucial role of this residue in the allosteric regulation of CPSase activity (Delannay et al. 1999). Substitution of Thr1042 by Ileu greatly lowers the capacity of ornithine activation, but the enzyme is still sensitive to UMP and IMP, although to a lower extent (Delannay et al. 1999; Rochera et al. 2002; Pierrat et al. 2002). Similarly, substitution of Ser948 by Phe results in an enzyme that is insensitive to UMP and IMP, but still activated by ornithine, though again to a reduced extent (Delannay et al. 1999). As Thr1042 and Ser948 are located in spatially distinct regions of the enzyme, it is evident that the mutations have coupled effects on the activation and inhibition pathways of enzyme regulation. For a full description or overview of the different residues and molecular interactions involved in the binding of the different effectors, the reader is referred to the structural papers on wild-type and mutant CPSases (Thoden et al. 1997, 1998, 1999b, c, d).

CPSase is fundamentally a heterodimer (αβ) but this “monomeric” form readily converts to dimeric (αβ)_2_ and tetrameric (αβ)_4_ species depending on the presence of effector molecules (Powers et al. 1980; Anderson 1986). Ornithine, Mg^2+^ATP and potassium, all activators of the enzymatic reaction, promote the formation of the tetrameric species, whereas the enzyme exists essentially as a heterodimer (αβ) in the presence of the inhibitor UMP (Kim and Raushel 2001; Mora et al. 2002). Mutant studies indicated that dimer formation relies essentially on interactions of regulatory domains, whereas tetramers are formed by interaction of two dimers across their oligomerization domain, but noteworthy oligomerization per se has no effect on the regulation of CPSase activity (Mora et al. 2002).

## Conclusion

The unique position of CP as a precursor common to the biosynthesis of arginine and pyrimidines, combined with the observation that *E. coli* harbors only a single enzyme that produces all the CP for both pathways, raises the need for a complex regulation of its synthesis at both the level of enzyme synthesis and enzyme activity. Such a combination is desirable and even indispensable since regulation of enzyme synthesis and allosteric regulation of enzyme activity operate at a different time scale. Regulation of enzyme synthesis by inhibition of transcription initiation affects the first step of gene expression and hence avoids waste of energy in the production of unnecessary mRNA and protein molecules, whereas fast inhibition/activation of enzyme activity by allosteric effector molecules allows fast adaptation of CP production in function of the needs of both pathways. This is particularly important in the case of CP, a labile molecule that should not accumulate in the cell. CP decomposition would not only represent a waste of energy (two moles of ATP are required to produce one mole of CP) but also generates the highly toxic cyanate. At the transcriptional level, the dual role of CP in the *E. coli* metabolism is reflected in the differential control mechanisms of the tandem pair of promoters directing transcription of the *carAB* operon. The downstream promoter P2 is repressed by arginine-bound ArgR, whereas the upstream promoter P1 is regulated by various mechanisms and effectors from both the pyrimidine and purine biosynthetic pathways. This situation ensures that enough CP is synthesized for the arginine pathway in conditions where P1 is repressed and vice versa that enough CP is produced for de novo pyrimidine synthesis when P2 is shut down upon high arginine supply. The antagonistic effects of ornithine (activator) and UMP (inhibitor) on CPSase activity and the consumption of CP as a substrate by the two carbamoylating enzymes (OTCase, ATCase) involved in the synthesis of arginine and UMP further ensure this delicate balance between CP production and CP consumption in function of the cellular requirements for both pathways. Much of the reaction mechanisms and their regulation, the enzyme structure and the protection of the highly labile intermediates in the production of CP could be solved by a combination of enzymatic, biochemical, genetic and structural studies with wild-type and mutant enzymes. These studies also revealed the presence of three distantly situated active sites connected through a 96Å internal tunnel. The study of transcriptional regulation of the *carAB* operon has revealed a complex interplay of several allosteric DNA-binding transcription factors using effector molecules from three different pathways (arginine, pyrimidines, purines), nucleoid-associated factors (NAPs), and trigger enzymes. Furthermore, DNA topology changes, UTP-dependent RNAP stuttering and stringent control by the alarmone ppGpp are involved as well. In this respect, regulation of CP production is an interesting example of the combined use of different gene regulatory mechanisms and effector molecules to coordinate the synthesis of a single metabolite involved in two distinct pathways. It also illustrates the diversity, flexibility and versatility of bacterial regulatory mechanisms. However, even though numerous players have been identified in the control of *carAB* transcription and CPSase activity, the mechanisms are not fully elucidated and much remains to be discovered on their interplay. ‘Avis aux amateurs’.
